# Multi-method approach effectively identifies and engages children impacted by parental substance misuse into school-based prevention services

**DOI:** 10.3389/fpubh.2024.1433917

**Published:** 2024-12-24

**Authors:** Julie Wood Merchant, Jessica Snell-Johns

**Affiliations:** ^1^Behavioral Health Services, Frederick County Health Department, Frederick, MD, United States; ^2^Promoting Positive Change, LLC, Annapolis, MD, United States

**Keywords:** parent substance abuse and dependency, adverse childhood experiences, screening, school mental health, at risk students, substance use prevention programs, evidence-based prevention programs (EBP), children of alcoholic parents

## Abstract

Children living in households where parents or caregiving adults misuse substances face significant risk academically, socially, physically, and emotionally. An estimated 12% or more of U.S. children lived with an adult with past-year substance use problems in 2009–2014. Engaging this high-need, underserved population in targeted prevention services is a public health imperative, requiring children first be identified. School-based services reduce access barriers and promote equitable access, providing a model that can address the scope and significance of parental substance misuse (PSM) on children. A review of published studies for this population revealed a lack of information regarding identification strategies and their relative effectiveness. This article uses data from a 2010–2020 field-based evaluation to analyze the performance of the Kids Like Us (KLU) program’s manualized approach to identifying and engaging elementary students impacted by PSM into its school-based program. KLU, a program of the Frederick County Health Department (Maryland, U.S.), is implemented in collaboration with public school counselors. KLU’s multi-method approach achieves universal prevention outcomes while simultaneously providing parent, self, school counselor, and community referral options. Over the 10-year study, 537 students were identified with a notable 83% of referred students completing 75–100% of sessions offered. Parent referral, a strategy not included in any reviewed studies, contributed the highest percentage of referrals (44% in response to a student take-home letter alone), followed by self (18%), school counselor (13%), and other/a combination (24%). KLU engaged students across varying school settings, sizes, and socioeconomics. Chi-square analysis of gender and ethnicity found no significant differences. Referral outcome and school counselor data results highlight the benefits of KLU’s multi-method, multi-source approach, including its ease of replication. KLU’s approach holds promise for addressing the public health crisis of children impacted by PSM. Study results highlight the need for policy changes including that U.S. and world alcohol and drug reports assess parent status. Additional research with a larger, more ethnically diverse population is recommended to examine the relationship between family and student demographics and referral strategies and sources.

## Introduction

Growing up in a household where parents or caregiving adults misuse substances is a highly significant and prevalent risk factor for children. Providing services to this underserved population requires that impacted children first be identified and referred. However, published studies regarding programs for this population provide few, if any, details regarding their methods for identifying and engaging the target population or the efficacy thereof. Using data from a 10-year, field-based evaluation study of a prevention program for children impacted by parental substance misuse (PSM), this article examines the Kids Like Us (KLU) program’s participant identification and engagement approach. Multiple methods and data sources are used to examine: the approach’s effectiveness in identifying students fitting the target population; the representativeness of students identified and referred compared to their school populations; the effectiveness across varying school sizes, community settings, and socio-economic factors; the differential contribution of referrals resulting from various referral sources; the extent to which the approach meets the population’s unique needs for confidentiality; the approach’s potential for replicability and increasing scale; and its effectiveness in also serving as a school and community-level prevention strategy.

In this article, the language used to describe the target population is “children living in households where parents misuse substances” and “children impacted by PSM.” This language, compared to other terms used historically in the literature (i.e., children of alcoholics, children of substance abusing parents, addiction, alcoholism, substance abuse), is conceptually important. “Household” aligns with the terminology put forth in the original U.S. Centers for Disease Control and Prevention Kaiser Permanente Adverse Childhood Experiences (ACE) Study (RRID:SCR_008382) (i.e., household substance abuse) (1) and is inclusive of diverse family structures. Children impacted by PSM is inclusive of children living in households where parents or caregiving adults are misusing substances and/or have a diagnosed substance use disorder (SUD). Defining the target population in this way aligns with a public health vs. a medical model, which would require diagnosing a parent with a SUD in order to identify the child as in a high-risk population. The exception to our use of this language is when referencing studies wherein authors used different terms. To acknowledge diverse family structures, we use the term parent to describe an adult responsible for the ongoing care of a child (e.g., biological parent, guardian, stepparent, foster parent, adoptive parent, relative, caregiver, partner of a parent, a household resident).

Children living in households where parents misuse substances face elevated risk for trauma-inducing experiences like witnessing violent behavior; observing and experiencing physical, emotional, or sexual abuse; neglect; loss (i.e., death, imprisonment, divorce, separation, foster placement); lack of a stable adult presence; knowledge of and/or experience of criminal activity; unclean home environments, including proximity to substance manufacture, use, and paraphernalia (2); and financial instability (3). It is not uncommon for children impacted by PSM to blame themselves for adult behaviors, feel a heightened sense of responsibility (i.e., for themselves, their parents, their siblings) (4), and operate under a family “no talk” rule (5). These experiences can amplify the emotional stress and physical consequences of trauma. In addition, stigma associated with PSM and family secrecy may lead children to internalize negative emotions (3) and prevent them from sharing what is happening in their family.

The original ACE Study (1) identified household substance abuse as the most prevalent (25.6%) of the seven ACEs, or potentially traumatic events, that can occur in childhood. Anda et al. (6) pointed out that the respondents reporting parental alcohol abuse were the most likely to report experiencing all seven ACEs. With a higher number of ACEs correlating with higher risk, youth living in homes where parents misuse substances stand out as being at the greatest risk among an already high-risk population (i.e., children experiencing ACEs). The 1998 ACEs study and a subsequent accumulation of evidence (7–9) establish broad agreement that growing up with PSM is linked to further increased risk for substantial negative outcomes including chronic health problems (2) and behavioral health problems, including SUDs (10, 11).

While the rates of alcohol and other drug use vary somewhat across countries, substance use and its relationship to other health disparities is a growing global public health concern. Worldwide, alcohol use increased over the past three decades and is expected to continue to do so, both in terms of prevalence and severity of use (12). An estimated 43% of those aged 15 years and older across the globe engaged in past-year alcohol use in 2016 (13). The World Health Organization (13) estimated that 45 to 60% or more of current alcohol consumers in the Russian Federation, European countries (e.g., Bulgaria, Poland, and Romania), several sub-Saharan African countries (e.g., Angola and Democratic Republic of the Congo), Australia, and countries in South America (e.g., Peru, Bolivia, Brazil) engaged in heavy episodic drinking (i.e., consuming 60 grams or 2.12 ounces or more of pure alcohol on one or more single occasions per month) in 2016. The National Center for Drug Abuse Statistics (14) reported that 60% of adults in the U.S. increased their alcohol consumption during the COVID-19 lock down.

The 2021 World Drug Report (15) reported that, in 2019, an estimated 5.5% of those ages 15–64 worldwide were past-year drug users (i.e., the 12 months prior to being surveyed), which is a 22% increase from 2010. In the U.S., the estimates of rates of adult past-year drug use are reported in two age groups: (a) young adults 18 to 25 and (b) adults 26 and older. Estimates of past-year illicit drug use among young adults increased from 37.5% in 2015 to 39.1% in 2019 and to 40.9% in 2022 (16, 17). Estimates of past-year illicit drug use among adults ages 26 or older increased from 14.6% in 2015 to 18.3% in 2019 to 23.7% in 2022 (16, 17).

Globally, 13% of those estimated past-year users of any drug, ages 15–64, suffered from drug use disorders in 2019 (15). Among young adults in the U.S., the 2019 rate (14.1%) of past-year SUD almost doubled to 27.8% in 2022. Among adults ages 26 and older in the U.S., the rate of past-year SUD in 2019 of 6.7% more than doubled to 16.6% in 2022 (16, 17). Individuals with a SUD and those in recovery faced increased challenges and vulnerability to severe COVID-19 illness and death (18) and had greatly reduced access to treatment (19, 20). Notable also in this context, is the reported statistically significant positive correlation between having children (i.e., being a parent or guardian) during the COVID-19 lockdown and increased alcohol consumption in Europe, Canada, and the U.S. (21).

Prevalence data regarding the percent of children worldwide or in the U.S. impacted by PSM is not available. Based on data from the combined 2009 to 2014 National Surveys on Drug Use and Health, Lipari and Van Horn (22) estimated that about 12% of U.S. children aged 17 or younger lived in households with at least one parent (biological, step, adoptive, or foster) who had past-year substance use that resulted in significant impairment. Based on data from the 2018 German Epidemiological Survey of Substance Abuse, Kraus et al. (23) estimated that about 5–9% of German children lived in households where at least one adult had an alcohol use disorder and about 1% of children lived in households where at least one adult had a disorder related to illicit drug use. These estimates do not include the identified and projected negative impacts of the COVID-19 pandemic on household substance use rates. This suggests that an even higher percentage of children have faced an increased risk of PSM and other ACEs (i.e., death of a parent, parental depression) (24, 25) as a result of the pandemic. The results of the 2021–2022 Maryland Youth Risk Behavior Survey/Youth Tobacco Survey indicate that 25.3% of high school youth and 18.3% of middle school youth reported having ever lived with someone who was having a problem with alcohol or drug use (26). Pre-pandemic levels are not available as this inquiry was new to the 2021–2022 version. Because the pandemic also resulted in decreased access to protective factors like caring relationships with adults and access to prosocial peers (27), the negative impacts of PSM have likely been greatly compounded.

The scope and risks associated with PSM, exacerbated by the pandemic, call on communities and schools to adopt strategies that effectively identify and engage this population of children so they can receive selective prevention, or prevention efforts designed for a group identified as being at higher risk for developing problems (28), in this case living in a household where parents misuse substances. By definition, selective prevention involves selecting or identifying individuals in a target population prior to providing prevention services. Similar to PSM, Reedz et al. (29) highlight how the failure to identify children impacted by parental mental illness has large implications for public health given that identification is a core requisite for intervention. Published studies of selective prevention efforts to address PSM demonstrate that participants experience positive results, such as improved coping skills, increased problem solving ability, enhanced social competency, and reduced isolation and loneliness (9, 30–36). For the past several decades, programs for this population have occasionally been offered in various settings such as SUD treatment centers, community agencies, and schools (37). However, results from the 2019 National Survey on Drug Use and Health (RRID:SCR_007031) (16) indicate that less than 2% of adults received substance use treatment in the past year, highlighting the fact that efforts to reach impacted youth are extremely limited if dependent on adults accessing treatment. Even if adult access to treatment increased greatly, most treatment is brief and thus, selective-prevention services for impacted children would be time-limited and not consistent with principles of effective prevention (38).

With an estimated 91% of U.S. youth attending public schools (39), schools are an obvious hub for providing core elements of a public health approach to children’s mental health (40). Through schools, children and families could have easy access to evidence-based programs and practices around a variety of mental and behavioral health topics, including the impact of PSM on children. School-based services, implemented in a confidential manner during the school day, decrease common access barriers like transportation, scheduling, parental readiness for change, and stigma of going to a treatment center (41).

Engaging children impacted by PSM requires thoughtful and ethical navigation through a complex intersection of circumstances and approaches informed by the unique needs of potential participants and their families. Compared to children in other high-risk groups where determinants of health are more readily assessed (i.e., obesity, low reading levels) or a matter of public record (e.g., death of a parent), children exposed to PSM are often uniquely hidden in plain sight of those who could readily provide support. Earning trust can be challenging for school personnel when family members work to ameliorate the associated shame and stigma of PSM through denial, minimization, and secrecy (42). Families may fear their child’s participation could result in further negative consequences, such as disclosure of illegal activities, concerns about confidentiality (8), or losing custody of their children (43). The children themselves may be simply unaware of or unable to name what is happening in their family (44) or may avoid disclosure at school (8). Even when educators are specifically trained on how PSM can impact children, student behavior may not elicit referral.

This article introduces an innovative approach used by the KLU program for nearly two decades to identify, enroll, and engage students impacted by PMS into its selective prevention program. We also examine how KLU’s approach to student identification and engagement aligns and contrasts with previously evaluated screening and engagement strategies through a review of approaches employed by other published programs.

The first school-based KLU groups started in 1989 in Frederick County (Maryland) Public Schools (FCPS) with fourth and fifth graders and expanded into secondary schools later that year. KLU includes two components: (a) a preventive intervention program providing four arms of direct service: school-based counseling groups implemented during the school day, referral and linkage, a summer day camp, and a community art show, and (b) a manualized process for identifying and referring students into the program. The focus of this article is evaluating the effectiveness of KLU’s manualized process for identifying and referring students into the program.

### Description of KLU’s multi-method, multi-source approach to student identification and referral into level one of program

The KLU identification and engagement approach involves time-targeted efforts led by the KLU-trained school counselor, including an annual classroom lesson, a take-home parent letter, follow-up and referral, as well as ongoing referral efforts. While the FCHD’s KLU counselors take the lead in implementing the school-based groups, collaborating FCPS school counselors play an important role in identifying and referring participants, maintaining participant confidentiality, and supporting successful program implementation. School counselor involvement in the identification and referral process, as well as in co-leading the school-based groups, is aligned with the school counselor role in providing education, assessment, direct interaction, linkage, and counseling. Because of the level of training of school counselors, at least in Maryland, school counselors are equipped to assess mental health needs, protect private health information, facilitate referrals, and collaborate with parents and community members to meet student needs. School counselor certification in Maryland requires a master’s degree in counseling or school guidance and counseling from a Council for Accreditation of Counseling and Related Educational Programs accredited program (45).

KLU further trains collaborating school counselors to use both time-targeted and ongoing identification and referral approaches using the 80 plus-page *KLU Handbook for School Counselors*. The KLU Handbook and training emphasize respect for all potential participants and families as well as best practices of school counselors, the Family Educational Rights and Privacy Act, and the Health Insurance Portability and Accountability Act (e.g., confidentiality of potential participants and their household members, not asking direct questions about individuals who misuse substances in the household). The KLU staff annually review, update, and improve the KLU Handbook and training based on their own observations, collaboration with school counselors and FCPS leadership, and evaluator recommendations based on analysis of school counselor feedback collected from an online survey administered every other year.

In Step One of KLU’s identification and referral approach, KLU-trained school counselors across Frederick County teach a 45-min prevention classroom lesson to all of the fourth grade classes at their school in the first term of the school year. This lesson is another way FCPS and FCHD partner in prevention. While serving as a strategy to prompt self-referral into KLU’s school-based groups, the classroom lesson is also universal prevention, or prevention aimed at the entire population (28), in this case, all fourth graders.

The fourth grade lesson includes a video and discussion about PSM. The lesson teaches how and when to seek help as well as coping strategies. The lesson also provides information about situations that children living in households where adults misuse substances are likely to encounter (i.e., one sibling taking care of another, parents arguing about the substance misuse, parents behaving erratically, children feeling worried or scared about what is happening). Much of this information applies to ways children can cope with other ACE’s (e.g., divorce, parent illness). The video and classroom lesson help students learn that if someone close to them misuses alcohol or other drugs it is not their fault and it is not their responsibility to change or fix it. The video reinforces non-stigmatizing messaging around SUD and people who misuse substances.

To reinforce the coping strategy of reaching out for help, students privately complete and return a *Request to See the Counselor* form during the lesson. This is an opportunity for students to self-identify as needing to see the school counselor. This form and process is the same or similar to how students across FCPS are accustomed to requesting individual time with their school counselors. The form allows students to choose a topic (e.g., today’s lesson, a playground issue) or to provide their own reason to speak with the school counselor. Students can also indicate they do not need to talk now. The school counselor explains to the entire class that students wanting to talk about the video, as well as students wanting to talk about other topics, can speak privately with the counselor. The counselor then collects the confidential forms. This approach protects student confidentiality by clarifying that if a student meets with the counselor, it does not necessarily mean the student is impacted by PSM.

Within a few days of the classroom lesson, the school counselor sends a letter (available in English and Spanish) home to parents of all the students from the classes receiving the lesson. KLU’s *Letter to Guardians of All Fourth Graders* provides critical information about SUD and prevention and recovery resources. The letter also introduces KLU as a confidential and positive opportunity to support students concerned about the substance use of someone close to them, explains that KLU participation does not depend on identifying whose substance use is of concern, and invites parents to refer their child by completing and returning the attached permission form in a confidential manner. The letter, provided on school letterhead, explains that the counseling program at the school is designed to support each student’s social and emotional health so that they can take advantage of their educational opportunities. It also reminds parents that children see the school counselor for a variety of reasons and that school counselors regularly teach classroom lessons, meet with students individually, and lead small groups as part of their role as a school counselor. This letter also invites parents to reach out to the school counselor with questions or for more information. Both the classroom lesson and the letter describe SUD as a disease and provide messaging intended to reduce stigma around substance use.

The methods utilized in Step One of the identification and referral process also serve as universal prevention aimed at educating teachers, parents, and all fourth graders about the impact of PSM and local and national resources for seeking help, including the Substance Abuse Mental Health Services Administration, the health department, KidsHealth.org, and the National Association of Children of Addiction. The video and follow up discussion are also specifically designed to increase compassion and concern for the often not discussed ACE of household substance abuse.

In Step Two, the school counselor meets privately with students who indicated they would like to talk about the classroom lesson. As guided by the KLU Handbook, the purpose of this individual meeting is to provide an opportunity for students to privately share thoughts and feelings about the video and lesson and to reinforce positive help seeking. Students may talk to their school counselor about any situation they wish, including the substance misuse of someone in their household; however, KLU trains school counselors to not ask about any home situation directly. This meeting creates an opportunity for the school counselor to determine whether or not a student’s particular situation meets the criteria for referral to KLU (i.e., someone close to them currently engaged in substance misuse in a way that worries or bothers them). If the school counselor thinks the student meets criteria, the counselor tells the student about the KLU group and gives the child the opportunity to self-refer. If the school counselor is unsure if the child meets criteria, the counselor is trained to reach out to the KLU program director to discuss the referral situation. If the school counselor thinks the student does not meet the criteria for KLU, or if a student who meets the criteria does not receive parent permission to participate, the school counselor, based on training and experience as a Maryland certified school counselor, determines appropriate next steps in supporting the student.

In Step Three, the school counselor calls parents who returned permission forms to ensure their understanding of KLU and to welcome the child into the program. In the final step, Step Four, school counselors compile a list of referrals, complete a confidential referral form for each student on the list, and provide these to the program to say KLU program director. The director then works closely with school counselors to confirm each student fits the target population. In schools with 70 students or less per grade level, KLU recommends school counselors conduct the above-described referral process with both fourth and fifth grade students every other year instead of every year. In order to identify enough students for a group, school counselors at schools with a total enrollment of less than 115 students, may include 3rd graders. Hereafter, student participants are referred to as fourth graders. KLU starts in fourth grade because of how the program fits into the overall FCPS health curriculum and fourth grade standards. Additionally, based on risk and protective factors (27), KLU aims to facilitate forming strong bonds with students before they transition into middle school and before youth would typically initiate experimental alcohol or drug use.

While most referrals come through the time-targeted efforts led by school counselors in the fall, KLU also invites ongoing referrals from school counselors and professional and non-professional community members. KLU promotes community member referral through a variety of means. These include a webpage with program and referral information, presenting an annual art show and public reception, meeting with local mental health providers, attending and speaking at school and community health fairs and meetings, and hosting full-day conferences about PSM featuring nationally known speakers, panels, and workshops. Community members, including families who may not have received or processed the fourth grade letter (e.g., did not read the letter, recently moved into the area) are encouraged and invited to refer impacted students to KLU by contacting either the KLU program director or the relevant school counselor.

To provide context for KLU’s selection and referral approach, we conducted a literature search for articles published between 1990 and March 2020 about how school-based, selective prevention programs similar to KLU go about identifying and engaging youth impacted by PMS. We focused on the steps taken by these programs to identify and refer these youth in grades three to 12 and the reported effectiveness of their identification, referral, and engagement strategies as compared to KLU’s approach. Using ERIC, PubMed.gov, Google, and Bing and the following keywords: children of substance abusing parents, children of alcoholics, children of drug users, household substance use problems, households with substance abuse, selective prevention programs, prevention programs for children from households with substance abuse, school-based prevention, recruitment, school-based mental health, and substance use disorders, we identified seven programs to review: (a) Students Together and Resourceful (46); (b) the Stress Management and Alcohol Awareness Program (34, 47); (c) Friends in Need (48); (d) Children Having Opportunities in Courage, Esteem, and Success (CHOICES) (33); (e) McNair and Arman’s (42) “small group model” program; (f) an earlier model of the School-Based Support Groups (we label version 1) (31); and (g) a later model of the School-Based Support Groups (we label version 2) (49). Our review focused on the steps taken by these programs to identify and refer youth in grades three to 12 and the reported effectiveness of their identification, referral, and engagement strategies as compared to KLU’s approach. The seven published programs represented various settings across the U.S. (i.e., suburban, inner city) and served different race/ethnic populations. Although statistical significance was not reported, the five programs reporting on gender suggested a difference in gender representation, with one program reporting a male majority participation rate and four reporting a majority female participation rate.

All seven programs described a single referral source of either self or school staff (e.g., school counselors, administrators, special educators, nurses, campus supervisors, or teachers). None of the programs described an option for parent or community member referral. In two reviewed programs (Students Together and Resourceful and Stress Management and Alcohol Awareness), the self-referral methods described overlap somewhat with KLU’s self-referral method. These programs begin with a school-based presentation of a video about PSM, and interested students attend a follow-up student assembly where the program is introduced. At the conclusion, the students who remain interested, take home a combined letter and parent consent form to participate in the group. The self-referral method used by the CHOICES program begins with a school counselor-taught universal prevention classroom lesson, including a video about a child overcoming concerns about parental alcoholism and a discussion. To this point, it is the most similar to KLU’s Step One of the time-targeted approach but diverges thereafter. As guided by the CHOICES program approach, the school counselor then solicits responses on a student screening questionnaire and follows-up privately with students whose responses indicated exposure to problems like those in the video and interest in participating in a group. School staff-referral strategies involved training adults to either administer student questionnaires or select students based on learning about how living in households where parents misuse substances impacts children.

While KLU developed its identification and referral process before reviewing strategies used by other school-based, selective prevention programs, KLU utilizes all of the strategies (i.e., video, classroom lesson, educating school staff about how PSM negatively impacts children, student survey) and referral sources (e.g., self, school counselor, and other school personnel) used by the other programs. However, KLU is unique in its multi-method and multi-source approach, particularly its use of parent and community referral sources.

## Materials and methods

This study focused on results from a 10-year, field-based evaluation of KLU’s manualized approach to selecting and referring fourth grade students impacted by PSM into its school-based, prevention program. Adhering to the methods and protocols as reviewed (full review) and approved by the Maryland Department of Health Institutional Review Board, this study used data from program records, referral forms, school records, personal communication with FCPS’s Supervisor of Facilities and Planning, and an every-other-year feedback survey of collaborating school counselors. Each of these data sources and methods is detailed below.

### Program records

KLU stores program records in a confidential Excel database. For this article, we examined data regarding participating schools, the number of program sessions offered, and student program attendance. We entered de-identified information into a confidential SPSS file to track how many referred students were enrolled, engaged, and completed the Level One group meetings offered during the referral year. A student was coded as *referred* into the program once KLU received a completed referral form. A referred student was considered *enrolled* into the program once KLU received parent consent and the student was determined to be an appropriate referral based on the target population definition. An enrolled student was considered *engaged* once the student attended at least one school-based session, and an engaged student was coded as having *completed* the program if the student attended at least 75% of the Level One group meetings offered during the referral year.

### Referral forms

The KLU referral form documents the information needed for a child to be enrolled and engaged into the KLU school-based prevention program. The person completing the form indicates how each referral fits the target population. Once received by the FCHD, the KLU program director reviews and makes a final decision about each referral.

To increase ease of use and decrease confusion, KLU has two slightly different versions of the referral form. One version is for collaborating school counselors and the other is for community-based referrals. While in both versions the person completing the form provides their own name and contact information, KLU needs and asks for fewer contact details from the collaborating school counselor than from community members. In both versions, the person completing the form provides the date of the referral and the following information about the student being referred: name, grade level, school, race/ethnicity (i.e., African American/Black, Asian, Hispanic/Latino, American Indian or Alaska Native, Caucasian or White, Native Hawaiian or Other Pacific Islander, or more than one race), gender (female or male), and date of birth. KLU asks school counselors to provide demographic information based on the student’s official school records, community members (e.g., social workers, therapists) to base this on information collected during their own intake process, and family members to base this on personal information. Notably, over the 10-year period of data collection, the guidelines, definitions, and categories for race/ethnicity and sex/gender changed. In this article, the methods and results are presented using the language used in 2010, when data collection began.

The target population is students directly and currently (within the past six months) impacted by PSM. The most common situations in which this would occur are: a child living in a household or regularly visiting a household where at least one parent is engaged in problematic substance use, a parent has been diagnosed with a SUD, or a parent is newly in treatment or recovery from a SUD. Other ways children are directly and currently impacted by PSM are collaboratively considered on a case by case basis between the referring party and the KLU program director. Both versions of the referral form also pose three questions used to confirm the student fits the target population and is appropriate for the group: (a) Does child have current, direct exposure to parent/caregiver substance abuse? (yes, no, or unsure, and if no or unsure, contact the KLU program director prior to completing this form); (b) Describe how this student meets the target population; and (c) Please list attributes that will help this student be a productive group member (i.e., seeking help, interested in receiving or giving peer support). KLU uses the third question to promote engagement and completion rather than a reason to decline enrollment. The answers to these questions and, in some cases, conversation between the referral source and KLU, result in a final list of referred students.

The main difference between the two referral form versions is the number of options for referral source. Both forms offered these seven options: community member (mental health professional, teacher, neighbor, friend, other family member, etc.); parent/guardian request; KLU staff; school counselor and student self-referral (not related to the time-targeted approach in the Handbook); do not know; and other (please describe). The time-targeted process enumerated in the KLU Handbook adds three additional referral source options to the school counselor version of the form: (a) Parent/guardian referred via permission letter sent home to guardians of all fourth graders, (b) Student referred from individual meeting with school counselor requested by the student on the Request to See the Counselor form provided in the classroom lesson, and (c) Other referred from individual meeting with school counselor lesson not requested by the student on the Request to See the Counselor form provided in the classroom lesson. For purposes of analysis, we combined five of the uncommon referral sources into one category and added a category for those referrals that came from both parent letter and self, resulting in six referral sources. Table 1 presents the time-targeted and ongoing referral methods, referral sources, and the six condensed coding options used in this study for analysis: *parent letter*, *self*, *parent letter and self, school counselor*, *other knowledge of the program – combined, and source not known.* The school counselor version of the KLU referral form also provides check boxes for KLU to track the outcome of appropriate referrals selecting from the following options: enrolled, appropriate referral and parent permission given; not enrolled, appropriate referral, but parent would not give permission; not enrolled, appropriate referral, but program was full; and not enrolled, appropriate referral, but program not available at the school. Data regarding the not-enrolled subcategory was inconsistently documented. KLU confidentially stores de-identified referral form data from the 2010/11 school year through the 2019/20 school year in SPSS.

**Table 1:** Referral form coding options, descriptions of each referral method and source, and corresponding analysis categories.

Coding options on referral form	Description of referral method and source	Analysis categories
Referrals resulting from time-targeted referral efforts led by KLU-trained school counselor		
Parent/guardian referred student via Step One *Permission Letter for Guardians of All 4th Graders*	Parent completed, signed, and submitted the permission portion of the letter sent home with all fourth graders from participating schools, and the school counselor conducted the follow-up phone call.	Parent letter
Student self-referred via Step Two individual meeting with school counselor requested by the student	Student asked to talk about the lesson on the *Request to See Counselor Form* during the classroom lesson, and the school counselor followed up.	Self
Parent/guardian referred student via Step One *Permission Letter for Guardians of All 4th Graders* and student self-referred via Step Two individual meeting with school counselor requested by the student	Parent completed, signed, and submitted the permission portion of the letter sent home with all fourth graders from participating schools, and the school counselor conducted the follow-up phone call. The student also asked to talk about the lesson on the *Request to See Counselor Form* during the classroom lesson, and the school counselor followed up.	Parent letter and self
Referrals resulting from ongoing referral efforts		
School counselor	School counselor refers based on awareness of student fitting target population but not directly related to the KLU targeted referral efforts.	School counselor
Student self-referral request	Student requests to participate outside of the time when the classroom lesson is conducted.	Other knowledge of program
Parent/guardian request	Parent/guardian requests for child to participate not directly related to take-home letter.	Other knowledge of program
Professional referral (i.e., mental health provider, administrator, teacher)	School professional (other than school counselor) or community professional refers based on knowledge of KLU and student needs.	Other knowledge of program
Kids Like Us staff	KLU staff refers based on knowledge of a student’s need (e.g., sibling involvement).	Other knowledge of program
Non-professional (e. g. community member)	Non-health professional community members refer based on general knowledge of KLU.	Other knowledge of program
Other (with a blank for description)	Indicates a source not previously described.	Other knowledge of program
Unsure	Source not known.	Source not known

### School records

We retrieved school-level student enrollment data, the percent of students receiving free and reduced meals, and demographic data by school from the Maryland State Department of Education (50). Because school demographics can change based on the specific cohort of students and county-level changes, we selected the 2018/19 school year as the most representative because it was the largest referral year during the 10-year study period. For each school referring students, we examined the demographic data for referred students and compared this with demographic data for all students in the corresponding grade levels.

### Personal communication with FCPS’s Supervisor of Facilities and Planning

We retrieved school-level community setting data from the FCPS Supervisor of Facilities and Planning, (E. Pasierb, personal communication, March 15, 2021). Each referring school’s location was identified as being in one of the four types of residential settings in the county: (a) urban areas (i.e., incorporated cities with more than 50,000 residents), (b) small cities/towns (i.e., incorporated towns/cities of less than 50,000 residents), (c) unincorporated growth areas (i.e., characterized by expanding housing development along commuter highways to Baltimore and Washington, DC), and (d) non-growth areas (i.e., rural areas, farms, and unincorporated communities). This information provided the four categories used to examine whether KLU’s referral and engagement approach is equally effective in producing referrals across community settings.

### School counselor feedback survey

Since 2013, KLU, in collaboration with FCPS, has administered an online School Counselor Feedback Survey every other year to school counselors from all schools collaborating with KLU. The survey includes several items asking school counselors to provide their perspectives on various aspects of the identification and referral process. Based on recommendations from the evaluation team, the survey items changed slightly over administrations. School counselors completed the School Counselor Feedback Survey during a designated time-period at their convenience and in an anonymous and voluntary fashion. This article only includes results from the 2018/19 administration; however, 2012/13, 2014/15, and 2016/17 surveys were conducted in essentially the same manner, and results were equally positive in nature with many of the same counselors participating in each survey administration. Because the focus of this article is on identification and engagement of elementary school students, results from middle and high school counselors are not included. In spring 2019, the FCPS Supervisor of Behavioral Health and Student Services sent an email with a link asking collaborating school counselors to complete the anonymous, online survey within the designated 2-week window. Eleven days after the initial request, the Supervisor sent a reminder email. The survey was closed three weeks after the initial request. The 2018/19 survey consisted of 18 items focused on the identification and referral process, school counselor ratings regarding their role in implementing KLU, barriers to implementation, and student and school outcomes counselors attribute to KLU implementation. Twenty school counselors (from 20 participating elementary schools) completed the 2018/19 KLU School Counselor Feedback Survey regarding the 2018/19 school year, resulting in a 100% response rate from elementary school counselors referring students into Level One.

## Results

### Effectiveness of identifying and enrolling students fitting the target population

#### Total number of students referred

From 2010/11 to 2019/20, 537 students were referred to Level One of KLU through use of KLU’s manualized identification approach. Between 2010/11 and 2019/20, there were 11,329 students in the corresponding collaborating schools and grade levels. Using a 95% attendance rate results in an estimated 10,763 students receiving the universal classroom lesson and Letter to Guardians of All Fourth Graders. This means that approximately 5% of all attending fourth graders were identified as fitting the target population.

One hundred percent of elementary school counselors completing the 2018/19 (*N* = 20) School Counselor Feedback Survey agreed that KLU’s identification and referral process identified students who otherwise may not have received prevention services. Ninety percent agreed the process effectively enrolled most, if not all, of the students fitting the target population into the program while 5 % disagreed, and 5 % “did not know.”

Of the county’s 35 public elementary schools, 24 referred students to KLU between 2010 and 2019. As shown in Table 2, the average number of elementary students referred each program year by school ranged from 1.8 to 6.8 students. Contributions by school to the total number of students referred into the Level One program ranged from less than 1% to 12%. Whether this difference in referral numbers is due to differences in how the approach was implemented at these schools or due to the number of children living in households where parents misuse substances is not known. Starting in 2016, there was an increase in KLU’s funding which led to adding elementary schools. During the 2010/11 school year, there were 30 students referred to Level One from six participating elementary schools. The largest referral year was the 2018/19 school year with 86 students referred from 18 schools.

**Table 2:** Referring elementary school participation by community setting: average number of referrals and # of years partnering with KLU from 2010 to 2019 compared to school-level demographics using 2018/19 data.

Level one referring schools (grade levels) from 2010 to 2019	Average # of referrals per school year 2010 to 2019	# of years school partnered with KLU 2010 to 2019	Total # of level one referrals from 2010 to 2019	Total school enrollment 2018/19 school year	% of students receiving free and reduced meals 2018/19
Urban, incorporated					
Elementary P (K-5)	6.8	5	34	690	35.1
Elementary Q (K-5)	3.4	5	17	517	100
Elementary J (K-5)	3.3	7	23	668	50
Elementary R (K-5)	3	1	3	732	82.9
Elementary S (K-5)	2.5	2	5	583	71.1
Elementary C (K-5)	2	2	4	627	68.5
Small city or town, incorporated					
Elementary M (3-5)	6.5	10	65	299	32.5
Elementary B (K-5)	5.1	9	46	727	33.2
Elementary I (K-5)	4.5	2	9	693	5.8
Elementary G (K-5)	4	3	12	613	21.3
Elementary T (3-5)	4	1	4	457	10.8
Elementary F (K-5)	1.8	6	11	247	32.8
Growth area, unincorporated					
Elementary E (K-5)	5.3	6	32	788	5.4
Elementary K (K-5)	4.9	9	44	602	38.6
Elementary N (K-5)	4.1	10	41	654	33.7
Elementary A (K-5)	4	9	36	650	37.1
Elementary U (K-5)	3.5	2	7	679	12.8
Elementary V (K-5)	3	5	15	475	33.1
Elementary W (K-5)	2.1	8	17	455	14.4
Non-growth area, unincorporated					
Elementary O (K-5)	6.5	2	13	494	20.3
Elementary H (K-5)	4.6	7	32	262	23.2
Elementary X (K-5)	2.9	8	23	184	31.6
Elementary D (K-5)	2.6	10	26	579	11
Elementary L (K-5)	2.6	7	18	99	43.4

K-5 = kindergarten through fifth grades. 3–5 = third through fifth grades.

#### Percent of students identified and referred who enrolled, engaged, and completed level one of the program

**Figure 1:**
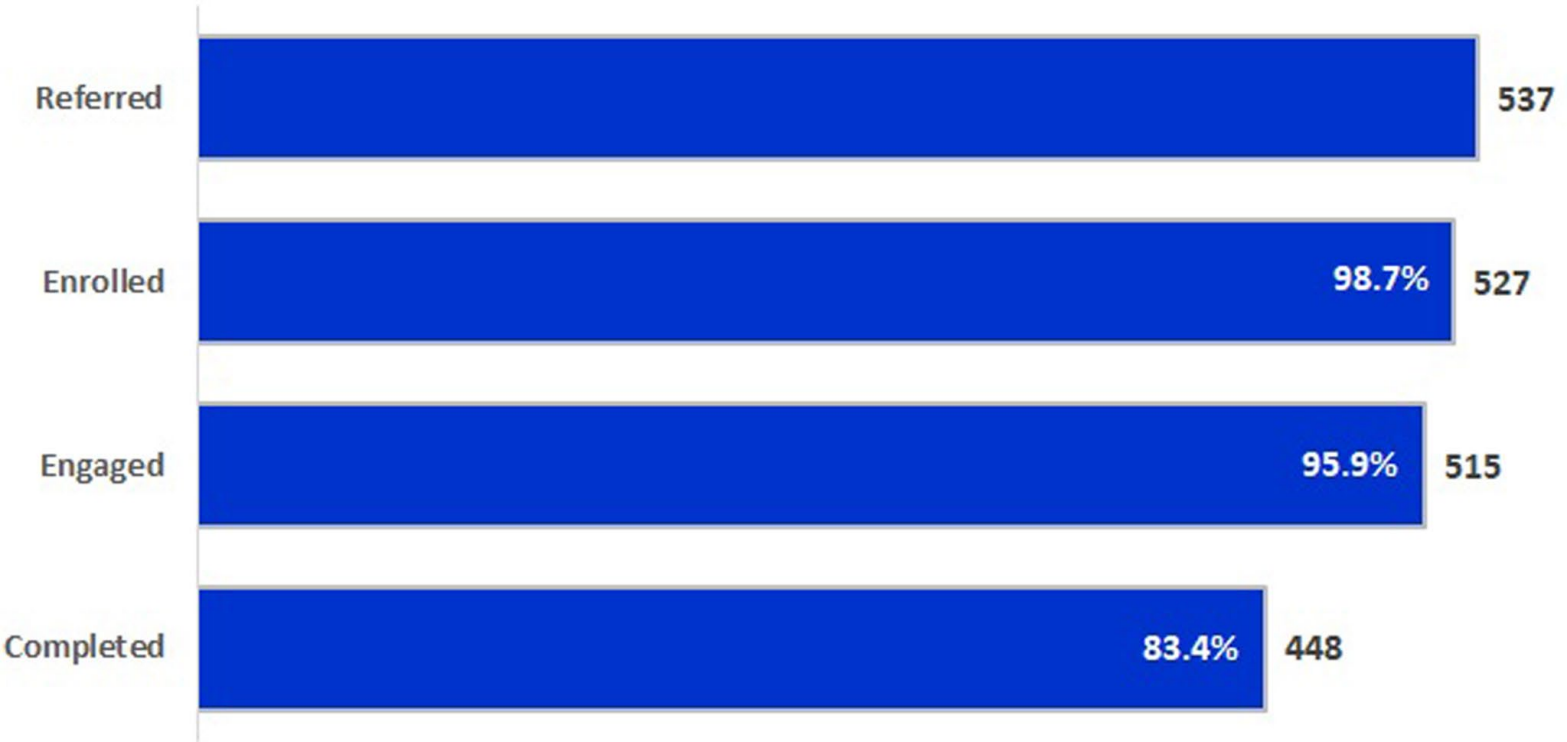
Percent and number of students referred, enrolled, engaged, and completing KLU level one from 2010–2019.

Figure 1 describes the number and percent of students referred, enrolled, engaged, and completing KLU Level One from 2010/11–2019/20. Ninety-eight percent (*n =* 527) of total referrals (*N* = 537) received parent permission and enrolled in the program, 96% (*n* = 515) engaged, and 83% (*n* = 448) completed at least 75% of the Level One group meetings. Of the ten students referred but not enrolled, nine were not appropriate referrals and one did not receive parent permission to participate. Of the 11 students enrolled but not engaged, six moved prior to the program start date and five for undocumented reasons. Of the 68 students engaged that did not complete at least 75% of the Level One group meetings offered during the referral year, 51 did not complete due to individual circumstances (e.g., moving away, low attendance), and 17 were unable to complete the Level One program related to system issues (e.g., when the group was too small to continue or was discontinued because of the pandemic).

#### Referral percentages across sex/gender and racial/ethnic groups

The sex/gender and race/ethnicity of referred students is examined in order to determine the effectiveness of KLU’s identification and referral approach in equitably reaching the target population. Data regarding adult past-year use suggests that children of both genders and racial/ethnic identities are vulnerable to the ACE of PSM. Thus, a difference in referral across groups could suggest a bias or limitation in the approach’s ability to reach the target population.

##### Sex/Gender

Fifty-five percent of the total of 537 program referrals were female, and 45% were male. In 2018/19, across the 18 elementary schools, considering all of the students in the corresponding grade levels, 52% were male and 48% were female. 2018/19 program participants were 57% female and 43% male. Using a one sample t-test, no statistically significant difference was found between the number of female and male students in the school vs. in the group, suggesting the identification approach is equally effective at identifying female and male students.

##### Race/Ethnicity

Of the 537 students referred, 70% were White, 14% African American/Black, 7% two or more races/ethnicities, 7% Hispanic/Latino, 1% of unknown race/ethnicity, <1% American Indian or Alaska Native students, <1% Asian, and <1% Native Hawaiian or other Pacific Islander. In the corresponding grades levels across the 18 elementary schools participating in 2018/19, ethnic/racial diversity ranged from 7 to 94% non-White. Due to the small sample size, we grouped non-White students for this analysis. Using a one sample t-test, no statistically significant difference was found between the number of White and non-White students in the schools vs. in the groups. Although the sample size is limited, this implies the school and KLU group numbers were demographically similar. Table 3 presents non-grouped race/ethnicity data, comparing the number of students in pertinent grade levels by each race/ethnicity in the 2018/19 participating schools with the number of students referred to Level One in 2018/19 by each race/ethnicity.

**Table 3:** Race/ethnicity of students attending vs. referred in participating schools and grade levels using 2018/19 school and program data.

Elementary schools and grade levels of referrals	All	White	African American/ Black	Hispanic/ Latino	Asian	2 or more races/ethnicities
	# of students in corresponding school grade levels *(# of students referred)*					
Totals reported	2006 (*86)*	--1092 *(60)*	--261 *(11)*	--287 *(8)*	*--10 (2)*	--21 *(5)*
Elementary A 4^th^	126 *(3)*	54 *(2)*	35 *(0)*	24 *(1)*	*-- (0)*	13 *(0)*
Elementary B 4^th^	104 *(6)*	78 *(5)*	9 *(0)*	9 *(0)*	-- *(0)*	8 *(1)*
Elementary C 4^th^	93 *(4)*	-- *(1)*	-- *(2)*	-- *(1)*	-- *(0)*	-- *(0)*
Elementary D 5^th^	91 *(3)*	70 *(3)*	-- *(0)*	-- *(0)*	-- *(0)*	-- *(0)*
Elementary E 4^th^	135 *(4)*	102 *(4)*	-- *(0)*	12 *(0)*	-- *(0)*	-- *(0)*
Elementary F 4^th^	47 *(3)*	41 *(2)*	-- *(0)*	-- *(0)*	-- *(0)*	-- *(1)*
Elementary G 4^th^	108 *(3)*	81 *(3)*	11 *(0)*	-- *(0)*	-- *(0)*	-- *(0)*
Elementary H 4^th^	45 *(2)*	42 *(0)*	-- *(2)*	-- *(0)*	-- *(0)*	-- *(0)*
Elementary I 4^th^	127 *(4)*	104 *(4)*	-- *(0)*	-- *(0)*	-- *(0)*	-- *(0)*
Elementary J 5^th^	101 *(4)*	41 *(2)*	22 *(1)*	25 *(1)*	-- *(0)*	-- *(0)*
Elementary K 4^th^	121 (5)	45 *(2)*	36 *(3)*	23 *(0)*	-- *(0)*	-- *(0)*
Elementary L 3^rd^ - 5^th^	63 *(4)*	56 *(4)*	-- *(0)*	-- *(0)*	-- *(0)*	-- *(0)*
Elementary M 4^th^	99 *(15)*	89 *(14)*	-- *(1)*	-- *(0)*	-- *(0)*	-- *(0)*
Elementary N 4^th^	138 *(5)*	67 *(4)*	33 *(1)*	22 *(0)*	10 *(0)*	-- *(0)*
Elementary O 4^th^ - 5^th^	113 *(7)*	85 *(7)*	-- *(0)*	--10 *(0)*	-- *(0)*	-- *(0)*
Elementary P 4^TH^	120 *(5)*	51 *(0)*	32 *(0)*	21 *(1)*	-- *(1)*	-- *(3)*
Elementary Q 4^th^ - 5^th^	218 *(4)*	-- *(0)*	50 *(0)*	130 *(3)*	-- *(1)*	-- *(0)*
Elementary R 4^TH^ -5^TH^	157 *(5)*	86 *(3)*	33 *(1)*	--11 *(1)*	-- *(0)*	-- *(0)*

*Note.* -- indicates exact count not available for 2018/19. These data are unavailable for students in relevant grade(s) because there were either <10 students or the percentage was either ≤5 or ≥95; therefore, the corresponding counts were suppressed to protect the identities, privacy, and personal information of individuals, or for an unspecified reason (Maryland State Department of Education, n.d.). Data on American Indian or Alaska Native and Native Hawaiian or Other Pacific Islander students are absent from this table because KLU received no referrals for these students in 2018/19. Consequently, the number for total reported is lower than the actual total number of students when examined by race/ethnicity.

#### Referral percentages across varying school sizes, community settings, and socio-economic factors

**Figure 2:**
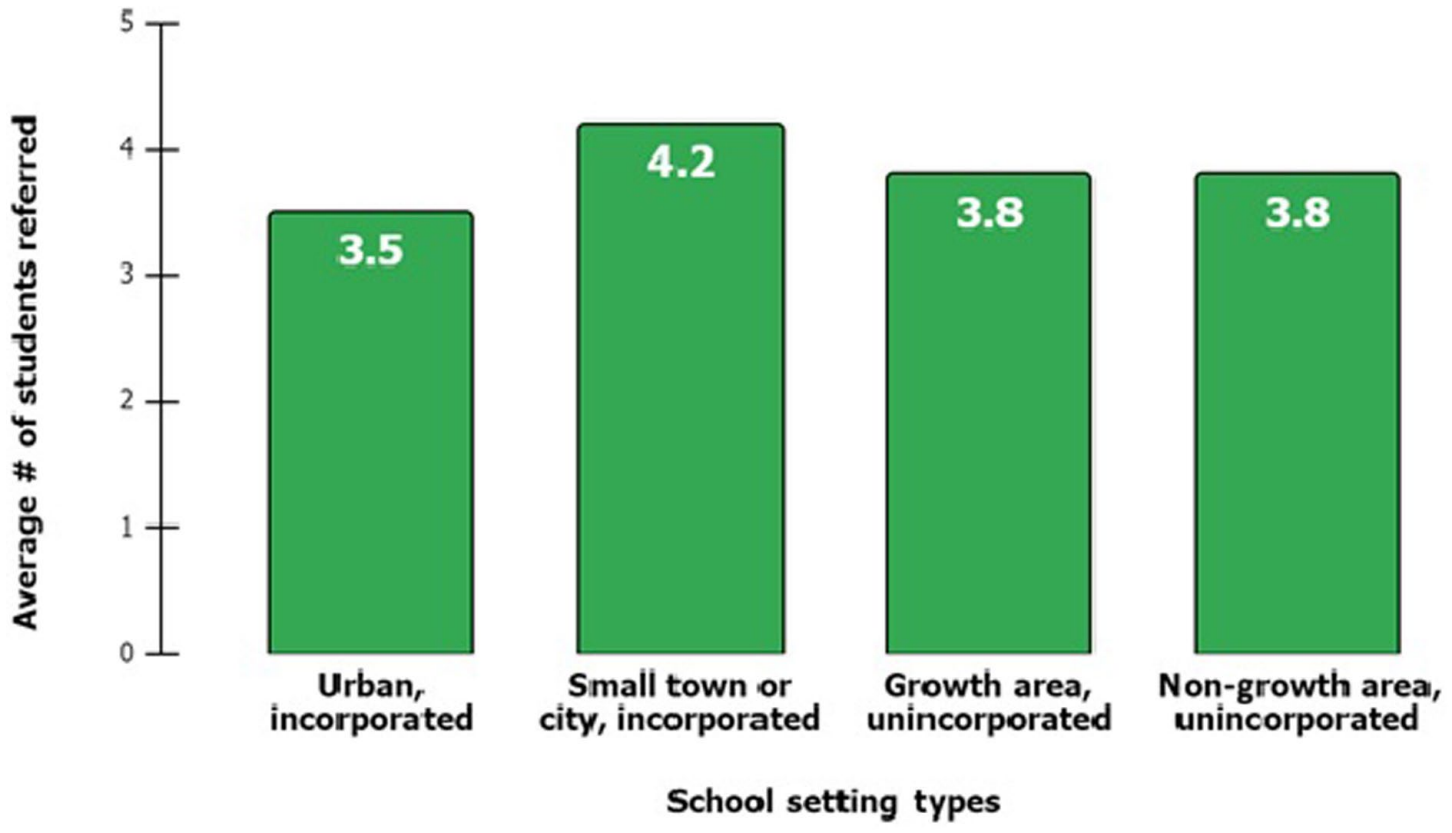
Average number of students referred annually by school setting.

Figure 2 shows the average number of students referred each year ranged from 3.5 to 4.2 students, suggesting that KLU’s approach is effective across various settings. This seems to indicate that school setting does not predict identification success. The average number of students referred each year ranged from 3.5 to 4.2 students, suggesting that KLU’s approach is effective across various settings indicating that school setting does not predict identification success. The total school enrollment in collaborating elementary schools varied in 2018/19 from 99 to 788. In that same school year, the percent of students in collaborating elementary schools qualifying for free and reduced meals ranged from 6 to 100%. Table 2 illustrates KLU’s success in engaging students across Frederick County’s range of school sizes, community settings, and percent of students receiving free and reduced meals.

### Differential contribution across referral sources

**Figure 3:**
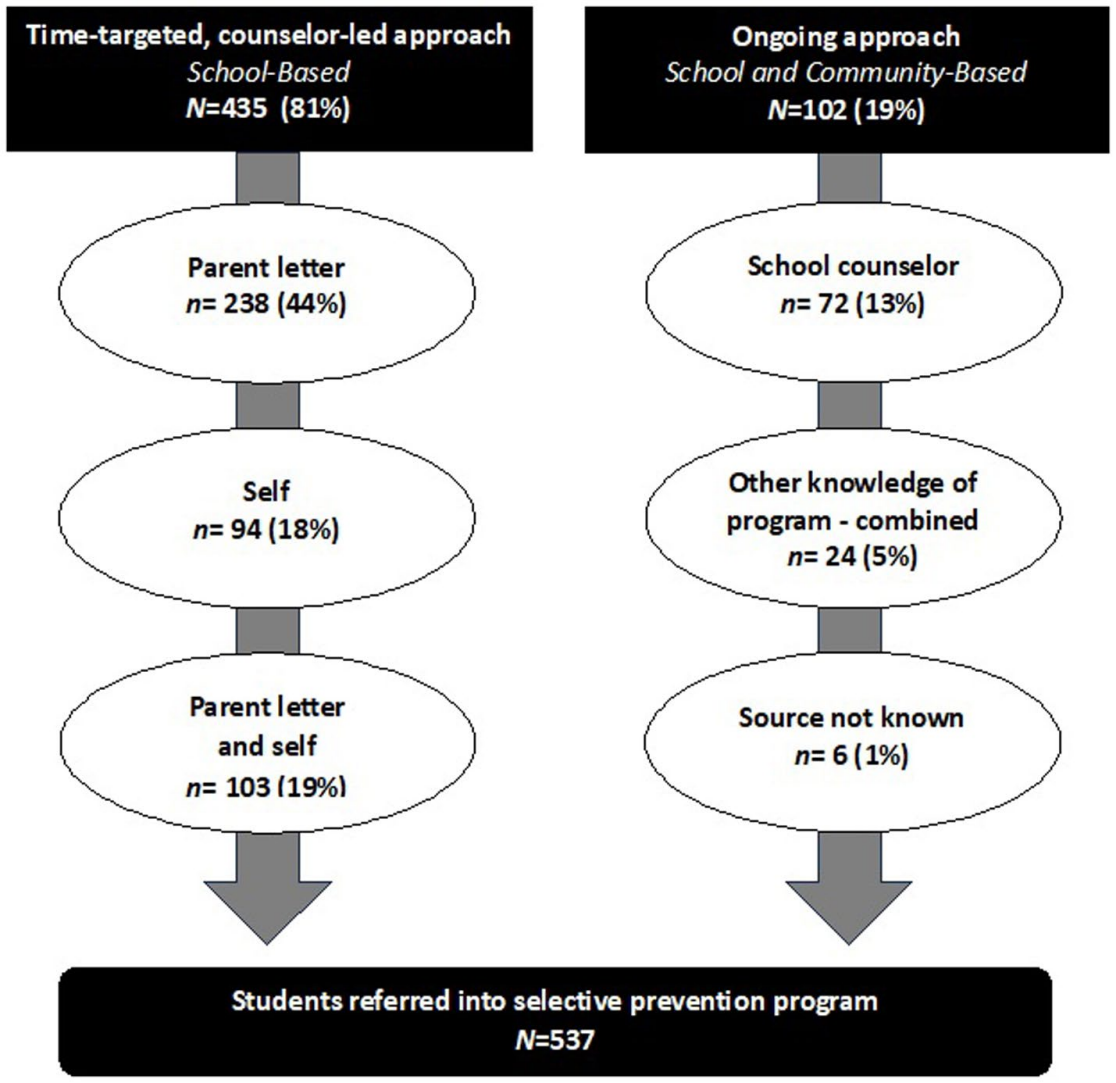
Referral source and method resulting in identification and referral into school-based selective prevention program.

Figure 3 shows the percent of referrals by source. Parent referral, a strategy not included in any reviewed studies, contributed the highest percentage of referrals (44%, *n* = 238) in response to a student take-home letter alone), self (18%, *n* = 94), school counselor (13%, *n* = 70), other/a combination (24%, *n* = 129), and not known (1%, *n* = 6).

The time-targeted counselor-led approach resulted in 81% (*n* = 435) of all referrals compared to 19% (*n* = 102) from the ongoing approach. Eighty-two percent (*n* = 238) of referrals resulting from the time-targeted approach were from the parent letter, either alone or when the student also self-referred. Of note is that 10 of the 238 students in the parent letter referred group were also school counselor referred, suggesting that the school counselor had a role in these referrals. Over 80% (*n* = 430) of referrals came as a result of the lesson-letter combination (i.e., referrals came either solely from parents giving participation consent from a letter sent home with students after the classroom lesson, students asking to speak to the counselor after the classroom lesson, or a combination of the two).

### Relationship between referral source and student sex/gender and race/ethnicity

Referral source data was analyzed by gender using a chi-square analysis at the 95% confidence level. No significant differences were found by sex/gender in the overall likelihood of referral within each referral source suggesting there is no relationship between sex/gender and referral source.

Referral sources were analyzed by race/ethnicity using a chi-square analysis at the 95% confidence level. No statistically significant differences were found, suggesting that a particular referral source was not better for one group than another. However, due to the small sample size within certain racial/ethnic groups and within certain referral sources, there was limited statistical power. Table 4 presents the distribution of racial/ethnic groups within each referral source (e.g., the number and percent of students from each racial/ethnic group that were referred by parent letter). Analysis of Table 4 suggests racial/ethnic distribution across most referral sources is consistent with the racial/ethnic distribution of referrals. However, since White students accounted for 85% (*n* = 61) of school counselor referrals compared to being just 70% (*n* = 373) of all referrals, Table 4 suggests that school counselors were more likely to refer White students than those of other race/ethnicity groups. Table 5 examines the distribution of referral sources within each race/ethnicity group (e.g., the number and percent of African American/Black students referred by referral source). Table 5 indicates that, across all ethnic groups, the most common referral source was the parent letter. There was a directional relationship suggesting that Hispanic/Latino students may be more likely to be referred through self-referral than other ethnic groups. Table 5 results also suggest there may be a greater likelihood for White students to be referred through their school counselor when compared to the referral rates of other ethnic groups.

**Table 4:** Distribution of race/ethnicity within each referral source.

Referral sources	Race/Ethnicity									
	White		African American/Black		Other^a^		Hispanic/Latino		Total	
	*n*	%	*n*	%	*n*	%	*n*	%	*n*	%
TOTAL	373	70%	77	15%	46	9%	36	7%	532	100%
Parent letter	160	68%	39	17%	21	9%	15	6%	235	100%
Self	64	68%	15	16%	5	5%	10	11%	94	100%
Parent letter and self (both)	69	68%	12	12%	13	13%	8	8%	102	100%
School counselor	61	85%	7	10%	3	4%	1	1%	72	100%
Other knowledge of program	15	65%	13	13%	3	13%	2	9%	23	100%
Source not known	4	67%	1	17%	1	17%	0	0%	6	100%

Five referred students of unknown ethnicity are not included in the table. In some cases, adding rounded percentages resulted in a sum slightly > 100%. ^a^Categories grouped for analysis: Asian, Native American, Native Hawaiian or other Pacific Islander, and more than 1 ethnicity.

**Table 5:** Referral source distribution within each race/ethnicity.

	Referral sources													
	Parent letter		Self		Parent letter and self (both)		School counselor		Other knowledge of program		Source not known		Totals	
	*n*	%	*n*	%	*n*	%	*n*	%	*n*	%	*n*	%	*n*	%
TOTAL	235	44%	94	18%	102	19%	72	14%	23	4%	6	1%	532	100%
White	160	43%	64	17%	69	19%	61	16%	15	4%	4	1%	373	100%
African American/Black	39	51%	15	19%	12	16%	7	9%	3	4%	1	1%	77	100%
Other^a^	21	46%	5	11%	13	28%	3	7%	3	7%	1	2%	46	100%
Hispanic/Latino	15	42%	10	28%	8	22%	1	3%	2	6%	0	0%	36	100%

Five referred students of unknown ethnicity are not included in the table. In some cases, adding rounded percentages resulted in a sum slightly > 100%. ^a^Other = categories grouped for analysis: Asian, Native American, Native Hawaiian or other Pacific Islander, and more than one ethnicity.

### Meeting population’s unique needs for confidentiality within a school-based setting

In the section of the KLU School Counselor Feedback Survey which asked collaborating elementary school counselors from participating schools to provide their perspectives on various aspects of the KLU identification and referral process, school counselors gave positive feedback. One hundred percent of school counselors agreed the process enrolled students whose parents likely would not have given permission for the program if the referral were handled in a less thoughtful manner, provided a fair (i.e., unbiased, objective) process for identifying and enrolling students into the program, helped families and students avoid stigma sometimes associated with substance abuse, and protected students from potential negative consequences of sharing that substance abuse is occurring in the family. Furthermore, 100% of school counselors (*N* = 20) agreed that KLU’s identification and referral process respected school counselor input, followed school regulations, and followed professional standards for school counselors.

### Identification approach also serving as universal prevention

While KLU targets and serves Frederick County youth in grades 4 to 12 whose lives are directly impacted by PSM, a central component to the identification process is a universal prevention strategy for fourth graders across the county. In the 2019/20 school year, the county had 35 elementary schools, and 19 (54%) of these elementary schools partnered with KLU. During the time-targeted efforts led by school counselors in the 2019/20 school year, 15 (43% of all) Frederick County elementary schools implemented the 45-min universal prevention classroom lesson with their fourth-grade classrooms. Based on the 2019/20 Frederick County average daily attendance rate of 95% (50), we estimate 46% (*n* = 1,353) of Frederick County’s fourth graders received the classroom lesson about children growing up in households where caregivers misuse substances, and their families received the information letter with local and national resources and information about SUD, recovery, and prevention. Of the 19 participating elementary schools in 2019/20, two schools with small class sizes used the time-targeted approach in their fourth and fifth grades and one school, with less than 100 students total, in grades three, four, and five. Three additional schools with small class sizes taught the universal prevention lesson with both fourth and fifth graders in 2018/19 and did not implement KLU’s identification and referral process in 2019/20, but conducted Level Two groups. One school did not have enough referrals (four or more) for a Level One group. A total of 63 fourth grade students, or 5% of all fourth graders estimated to have received the classroom lesson (1,353), were enrolled in the school-based, selective prevention aspect of KLU for the 2019/20 school year.

In addition to the results regarding implementation of the classroom lesson across the county, the KLU School Counselor Feedback Survey provides information regarding the perceived impact of the lesson. Eighty percent (*n* = 16) of elementary school counselors indicated that showing the video resulted in kids reaching out to share concerns, 5% (*n* = 1) said No, and 15% (*n* = 3) indicated this did not apply or they did not know. Ninety-five percent of elementary school counselors reported KLU helped raise awareness about the negative impact of family substance abuse on students, and 5% (*n* = 1) said it did not. Ninety-five (*n* = 19) percent of elementary school counselors also indicated KLU materials helped them be more attuned to substance use related issues for all students. Fifty-five percent (*n* = 11) of elementary school counselors indicated KLU helped raise awareness among school staff about problems associated with substance misuse, while 30% (*n* = 6) said they did not know, 10% (*n* = 2) said it was not applicable, and 5% disagreed. Another reported outcome of the universal prevention aspect to the KLU approach is that 90% (*n* = 18) of elementary school counselors agreed or strongly agreed that the process identified students who needed school counselor support even though they did not fit the KLU target population, 5% (*n* = 1) disagreed, and 5% (*n* = 1) said they did not know.

### Replicability and scalability

KLU’s identification and referral approach, applied over the 10-year period of the study (and in current use), reliably engages students into its Level One program. Over its 30 + −year program history, new principals have signed on, new schools have been adopted, and new counselors have been trained, with consistent and growing levels of support at the county level from both the health department and the public school system. KLU prioritizes program expansion in currently participating enrollment areas, or feeder patterns, and expands to further areas beginning at the elementary level where there is potential to engage middle and high schools as students age up through the program. KLU provides both the manual (i.e., *KLU Handbook for School Counselors*) and training to prospective school counselor collaborators to initiate strong, collaborative relationships and establish consistent administration. The manual and training provide guidelines for maintaining respect for potential participants and families, including confidentiality, and detail the steps of administering the time-targeted (i.e., classroom lesson plan, parent phone calls, answers to frequently asked questions) and ongoing identification and referral efforts. KLU teaches prospective school counselor collaborators, prior to their direct involvement, how to use the manual. The training is updated annually based on observations and feedback. As shown in Table 3, a few schools partnered with KLU during the entire study period, while others only partnered for one or two years. In only one situation was the reason for only one year of referral due to a discontinuation of participation, which in this case was due to a change in administration. In other cases, the number of years being less than ten years was due to a school joining the program at some point during the ten-year window, the school counselor not finding enough referrals for a group, or the school referring students every other year due to their small school size. Consistency of school participation and continued success with identifying students with high levels of enrollment, engagement, and program completion establishes that KLU’s approach is manageable and productive to replicate.

Decades of prevention research suggest often prevention programs found to be efficacious in the community setting do not maintain outcomes once implemented in school settings or are not successfully sustained (51). In contrast KLU and FCPS have collaborated successfully for over 35 years to identify children impacted by PSM and provide a multi-year, multi-strategy school-based prevention program. When asked to rate the extent to which any of the items from a list of possible barriers were a barrier to KLU implementation, 100% (*N* = 20) of the elementary school counselors indicated *not a barrier at all when asked about* lack of administrative support, lack of clarity about the school counselor’s role with KLU, and lack of communication from the KLU staff. When asked about lack of teacher support as a possible barrier 70% (*n* = 14) said *not a barrier at all*, 25% (*n* = 5) responded *a small barrier*, 5% (*n* = 1) responded *somewhat of a barrier*, and none responded *a large barrier.* Results regarding referral outcomes, ongoing school participation across program years, school sizes, student demographics, and community settings, combined with consistently positive survey results from school counselors regarding the effectiveness of the approach and their capacity to implement without barriers, indicates the ability of this approach to go to scale and be replicated. Replication and scale have already occurred within a large county, and materials are prepared for use in other counties or states.

## Discussion

### Identification and referral approach effective across range of participants and settings

This study investigates KLU’s approach to identifying students living in households with substance use problems in order to engage them into selective prevention services designed to address their unique needs. A major finding of this study is KLU’s success in effectively identifying 537 children living in households where parents misuse substances for its school-based prevention program. Feedback from collaborating school counselors indicates broad agreement that the process effectively enrolled most, if not all, of the students fitting the target population into the program. Many efforts to reach youth impacted by parental SUD have been group interventions connected to adult treatment programs. Unlike these and other efforts to engage children negatively impacted by PSM, KLU’s referral approach is not dependent on caregiver engagement in treatment, the diagnosis of a SUD, or requiring that families share tightly held information about family substance use. Given the estimate that 12% or more of U.S. children are impacted by PSM, and the estimate that 5% of fourth graders were identified, the program appears to be identifying 42% of the estimated total target population. Comparison of sex/gender and race/ethnicity of the referred students to those in the relevant grades at their schools revealed no statistical differences. This suggests that the KLU approach is promoting equitable access independent of sex/gender and race/ethnicity.

As with other approaches that were inclusive of children from minority and majority populations, analysis of KLU’s approach suggests it is effective with boys and girls and students of diverse race/ethnicity. Our results also indicate similar rates of effectiveness across all types of school settings including schools in urban areas, small cities/towns, and unincorporated commuter route growth areas, and non-growth areas, such as rural and agricultural areas. Likewise, participation was robust at schools with both high and low percentages of students receiving free and reduced lunches suggesting effectiveness in reaching students in various socio-economic situations. These outcomes indicate that KLU’s identification and engagement strategy is effective across a range of populations. Finding no positive correlation between the size of the school and the average number of students referred by year suggests the referral rate is impacted by other school-level and community-level variables. One possibility is the uneven distribution of school counselor caseloads from 99 to over 700 students. Other possible contributors are additional roles school counselors play in different schools (i.e., substitute administrator, behavior management, lunch, recess, bus duties), experience level of the school counselor with the referral process, trust level between the school and community, and possible differential rates of substance use.

KLU’s referral outcomes are consistent with what would be predicted given the at least modest referral success of other published selective-prevention programs for this target population using fewer referral sources and methods and not integrating parent-referral (32–34, 42, 46–49). Given that KLU’s approach integrates all sources and methods previously used, it makes sense the KLU approach is identifying 5% of fourth graders across a range of participants and school settings, among a population previously defined as hidden in plain sight and largely not served.

### Parents as effective source of referral

Another major finding from this study is that the method of showing students a video about PSM, sending home a parent letter about KLU and PSM, and allowing parents to refer their child was the largest single source of referral. This challenges what appears to be previously held assumptions about the validity and practicality of parent referral for this population. Our review of other school-based, selective prevention programs in peer-reviewed published studies demonstrated a reliance on self and school staff referrals. While the KLU approach to identification and referral includes self and school staff referrals, it adds the options of parent and community/other referrals.

When conducted in partnership with public schools, a parent referral letter is a free, easy-to-implement referral method with a notable absence in the literature regarding its use with other school-based programs. Relatives with custody of children due to neglect, absence, or death of a parent related to problematic substance use, non-using parents, parents newly in SUD recovery, and parents with untreated SUD may not only be open to the child participating in a program such as KLU, but also actively seeking support through the school or community. By not providing this easy option for referral, programs are likely missing a significant portion of the target population. Sending permission forms home with students is already a part of school culture (i.e., field trips, school pictures). Although incorporated into the *other knowledge of program – combined* referral category for analysis, nine students (2% of total referrals) were based solely on the parent knowledge of the program and reaching out to have their child referred. This accounted for more than one-third of the referral sources in this combined category, highlighting the value of the health department and school system partnership and the potential to facilitate community-based outreach through a largely school-based approach.

The value of parent referral in identifying this population is not surprising when considering the well-established role of parent engagement in all aspects of children’s health and well-being (52). It seems previous identification efforts may have been influenced by the assumption that parents who misuse substances misuse would not refer their children into prevention services for the impact of PSM. The results of this study challenge this assumption and demonstrate consistency with results from other types of programs regarding the importance of parent referral.

### Identification and referral approach also effective as universal prevention

The KLU fourth grade lesson (i.e., video and follow up discussion) is an integral step in identifying and referring impacted youth for selective prevention, but it also simultaneously provides important messaging about PSM for all students and school staff. KLU’s letter sent home by the school counselor informs interested families about KLU and how to refer their children and information about SUD, prevention, and recovery resources. In this way, KLU’s letter offers all families, including those who may not be ready to engage their child in KLU, critical and readily available SUD prevention and recovery resources. Use of videos in substance abuse prevention is well-established (36, 53). The Substance Abuse Mental Health Services Administration (54) recommends using videos to connect with the audience in implementing prevention strategies to effectively impact attitudes and change norms. Videos provide consistent messaging in an easily-digested form. Using a video to educate and promote de-stigmatizing language around substance misuse with the whole population of 4th graders and their teachers provides a way to change norms and attitudes about PSM. Our findings are also consistent with those of the reviewed programs reporting self-referral results following a universal prevention video about PSM (33, 34, 46, 47).

### The promise of school-community partnership to address the public health crisis of children impacted by PSM

In the U.S., public schools served 91% of youth in 2021 (39). Results from this study highlight the opportunity of school-based selective prevention efforts (37) and the promise of KLU’s approach to identifying and engaging one of the highest risk and least-served populations. KLU’s referral process also offers an opportunity for students, regardless of gender, race/ethnicity, residential area, and socio-economic status, to enroll successfully and receive year-round selective prevention programming that spans from elementary school to high school graduation. With 90% of the elementary school counselors that employed KLU’s identification and referral approach agreeing or strongly agreeing that the KLU referral process identified students who needed school counselor support even though they did not fit the KLU target population, it is clear there is value in the program that is missed if conducted in a community setting. Furthermore, KLU’s engagement approach facilitates communication between students and their school counselors, increasing access to mental health support and other health prevention and promotion services.

KLU is effectively identifying children living in households with PSM, achieving a critical first step in addressing the public health crisis of PSM. This identification and engagement success is not surprising given KLU’s integration of school-based strategies as well as parent and community referrals. Taking a public health approach is necessary to address health concerns as significant and prevalent as PSM. KLU’s approach utilizes cross-sector identification strategies, systematically addresses ACEs by identifying those impacted, and enables equitable access.

### Lessons learned

When the IRB-approved study was originally designed, the primary focus was on outcomes for KLU participants (e.g., sense of social support, coping skills). As the field-based evaluation study progressed, and after KLU was awarded a 2017 National Exemplary Award for Innovative Substance Abuse Prevention Programs, Practices, and Policies from the National Association of State Alcohol and Drug Abuse Directors, we became increasing aware of the innovation of KLU’s identification and engagement approach. While tracking the effectiveness of KLU’s identification and engagement strategies were always part of the study, we have now identified data that is relevant to the effectiveness of the approach that we did not track. For example, the referral process does not ask referral sources to document referrals not made because the parent would not grant permission for referral. Furthermore, not realizing the value of being able to examine each discrete and combined referral source, the referral form did not guide referral sources to document what they saw as the primary source of referral. Another lesson learned is to document the exact number of students present on the day of the classroom lesson and receiving the parent letter, allowing a more accurate analysis of those receiving the referral opportunity.

A final lesson learned is the importance of further evaluating fidelity to the manualized identification and referral process. While several aspects to handbook fidelity were assessed (e.g., use of the appropriate forms, implementation of the classroom lesson, sending home the parent letter), the quality of these efforts was not measured. Without this information, the extent to which the quality of the process (e.g., nature of the individual student meetings, counselor conversations with parents) influenced the number of referrals cannot be answered.

## Recommendations for future research

### Examine unique contribution of video as strategy for universal prevention and identification and referral into selective prevention program

While we believe the video in the classroom lesson is likely a contributing factor in the success of a child bringing the program and referral opportunity to a parent’s attention, the unique contribution of the video on self and parent referrals (i.e., because of how the video potentially influences the student’s conversations at home) needs to be examined. Also, we recommend that future studies focus on determining how many referrals were not made by school counselors or community members because of lack of processing the paperwork vs. parental refusal of the service for their child. By identifying the number of children fitting the target population whose parent does not provide consent at this point in the process, further work could be done to improve the referral strategy. Results from school counselors indicate that KLU’s identification and referral approach increases awareness and provides resources across the county, even if it did not identify impacted children, which it does.

### Learn more about parents who refer in response to letter and to school counselor outreach

Results indicate that sending home a letter to parents followed by school counselor outreach is a highly effective method for identifying and engaging children impacted by PSM. However, we need to learn more about this population of parents. We recommend additional research to explore whether the majority of parent referrals come from parents who view themselves or someone else, or both, as having a problematic relationship with substances. By learning more about how various populations of parents perceive the KLU referral process, we can potentially increase access to the target population. KLU has worked from the assumption that asking families to disclose who has the substance use problem would reduce referrals, but this needs further examination. This research could be conducted after students have been engaged through an optional follow up study.

### Conduct study with larger, more ethnically diverse population to examine relationship between family and student demographics and referral strategy

We may not have had the sample size needed to identify possible relationships between race/ethnicity and source of referral. The limited statistical power suggests that meaningful relationships could exist. For example, Hispanic/Latino children may be more likely to be self-referred from the classroom lesson than African American/Black or White children, supporting KLU’s practice of providing the parent letter sent home after the classroom lesson and community outreach materials in both Spanish and English. White students may be more likely to be referred through their school counselor than other ethnic groups. This could be due to a variety of factors (i.e., language barriers, lack of diversity of school counselors) that need to be explored in future studies. Another possibility for further examination is whether certain groups of students respond more readily to the opportunities for self-referral.

## Recommendations for future policy and practices

Based on our findings regarding the success of the KLU approach to identifying, referring, enrolling, and engaging children impacted by PSM, we recommend a manualized, multi-strategy approach like the one defined and used by KLU. Given the lack of evidence-based programs for children negatively impacted by PSM in clearinghouses and databases of programs, we recommend ACE-targeted programming and use of ACEs as search terms in clearinghouses and registries. Furthermore, because childcare can be a barrier to parents being able to complete residential SUD treatment, we recommend that search engines for SUD treatment and recovery services, include filters that more readily allow referring professionals, family members, and those with an SUD to easily determine which programs allow children to live with their parents during treatment and what, if any, services the program provides regarding the impact of the use on the children.

To fulfill a public health approach to addressing the critical situation of children impacted by PSM, we need clear data about how many of those with SUDs are parents and how parenting affects substance misuse. If national surveys examining the status of substance use and mental health provided this information in addition to information about demographics like race, age, and marital status, we could better address and examine the effectiveness of strategies meant to identify and treat SUDs among parents as a specific target population and provide effective, age-appropriate preventive intervention with their children. Furthermore, we recommend that future studies examine the interaction effect between demographics and referral sources with a larger sample size. Further examination could lead to better understanding of how/if various recruitment strategies apply across demographic groups evenly or if some strategies are more robust with certain demographic groups and how strategies could be enhanced to increase the potential to reach more of this underserved population.

To address the public health crisis of children living in homes where adults misuse substances, we recommend that school systems across the country adopt KLU’s approach to identification and referral. Even if schools are not able to offer a multi-session, multi-year program like KLU, the findings suggest KLU’s identification and engagement approach is an easily implemented strategy that utilizes existing roles and resources to successfully identify children experiencing one or more ACE’s, thus allowing schools to offer school and community-based resources to reduce risk and promote resiliency.

## Conclusion

KLU’s manualized, identification and referral approach was applied consistently over the course of the 10 years of study and reliably engaged students into its Level One program. Our results illustrate the efficacy of utilizing a combination of strategies, which include training school counselors to implement consistent practices county-wide year after year; supplying school counselors with information and resources; providing a universal prevention classroom lesson with a relevant video; supplying a parent letter with destigmatized messaging about substance misuse and SUDs across the school district; and allowing for parent, self, school counselor, and other community member referral. The KLU program has materials, handbooks, and evaluation tools so that the program is ready for prevention professionals to easily disseminate and replicate KLU’s identification and referral process. There is a tremendous need for programs like KLU that identify and support youth impacted by PSM and other ACEs. KLU’s readily adoptable approach holds promise for identifying and engaging children growing up with PSM, arguably the highest-risk and least served group of children in the U.S., so they can receive targeted, school-based prevention services.

## Data Availability

The datasets presented in this article are not readily available because of confidentiality requirements. Requests to access the datasets should be directed to the corresponding author.
